# A randomized, double-blind, crossover, placebo-controlled clinical trial to assess effects of the single ingestion of a tablet containing lactoferrin, lactoperoxidase, and glucose oxidase on oral malodor

**DOI:** 10.1186/s12903-016-0199-7

**Published:** 2016-03-22

**Authors:** Manabu Nakano, Eiju Shimizu, Hiroyuki Wakabayashi, Koji Yamauchi, Fumiaki Abe

**Affiliations:** Food Ingredients & Technology Institute, Morinaga Milk Industry Co., Ltd., 5-1-83 Higashihara, Zama, Kanagawa 252-8583 Japan; Shimizu Dental Clinic, 1066 Kamikobanamachi, Takasaki, 370-0077 Japan

**Keywords:** Oral malodor, Volatile sulfur compounds, Lactoferrin, Lactoperoxidase, Glucose oxidase, Randomized clinical trial

## Abstract

**Background:**

The main components of oral malodor have been identified as volatile sulfur compounds (VSCs) including hydrogen sulfide (H_2_S) and methyl mercaptan (CH_3_SH). VSCs also play an important role in the progression of periodontal disease. The aim of the present study was to assess the effects of the single ingestion of a tablet containing 20 mg of lactoferrin, 2.6 mg of lactoperoxidase, and 2.6 mg of glucose oxidase on VSCs in the mouth.

**Method:**

Subjects with VSCs greater than the olfactory threshold in their mouth air ingested a test or placebo tablet in two crossover phases. The concentrations of VSCs were monitored at baseline and 10 and 30 min after ingestion of the tablets using portable gas chromatography.

**Results:**

Thirty-nine subjects were included in the efficacy analysis based on a full analysis set (FAS). The concentrations of total VSCs and H_2_S at 10 min were significantly lower in the test group than in the placebo group (−0.246 log ng/10 ml [95 % CI −0.395 to −0.098], *P* = 0.002; −0.349 log ng/10 ml; 95 % CI −0.506 to −0.192; *P* < 0.001, respectively). In the subgroup analysis, a significant difference in the concentration of total VSCs between the groups was also observed when subjects were fractionated by sex (male or female) and age (20–55 or 56–65 years). The reducing effect on total VSCs positively correlated with the probing pocket depth (*P* = 0.035).

**Conclusions:**

These results suggest that the ingestion of a tablet containing lactoferrin, lactoperoxidase, and glucose oxidase has suppressive effects on oral malodor.

**Trial registration:**

This trial was registered with the University Hospital Medical Information Network Clinical Trial Registry (number: UMIN000015140, date of registration: 16/09/2014).

**Electronic supplementary material:**

The online version of this article (doi:10.1186/s12903-016-0199-7) contains supplementary material, which is available to authorized users.

## Background

Oral malodor is a common condition and a source of concern among a large number of people. When oral malodor is severe or long-standing, it may have a negative impact on self-confidence and social interactions [1]. A previous study indicated that oral malodor is the third most common reason, following dental caries and periodontal disease, to visit the dentist [2]. Volatile sulfur compounds (VSCs), mainly composed of hydrogen sulfide (H_2_S) and methyl mercaptan (CH_3_SH), are the main causes of oral malodor [3]. VSCs occur due to the metabolic degradation of sulfur-containing amino acids by the lyases of oral anaerobes [4]. A previous study reported a strong correlation between periodontal conditions and the concentrations of VSCs [4]. VSCs have also been identified as toxic agents that accelerate the progression of periodontal disease by increasing the permeability of the oral mucosa [5], suppressing the synthesis of collagen [6] and inhibiting the proliferation of osteoblasts [7].

Different treatment strategies including rinsing with antimicrobial agents have been proposed for the management of oral malodor [8, 9]. Although an antimicrobial mouth rinse containing chlorhexidine was previously shown to effectively suppress oral malodor [8], the use of chlorhexidine for extended periods of time has been associated with side effects including tooth and tongue staining and a bad taste [8, 10].

Lactoferrin (LF) and lactoperoxidase (LPO) are glycoproteins that are found in milk, saliva, and other exocrine secretions [11–14]. LF and LPO have been shown to exert antimicrobial effects against oral pathogens. LF exhibits antibacterial effects against periodontopathic bacteria, including *Porphyromonas gingivalis* and *Aggregatibacter actinomycetemcomitans* [12, 15, 16], and antibiofilm effects against *P. gingivalis* and *Prevotella intermedia* [17]. LPO catalyzes the hydrogen peroxide-dependent oxidation of thiocyanate (SCN^−^) to hypothiocyanite (OSCN^−^), which is a potent antimicrobial agent against bacteria, fungi, and viruses [13, 14]. This antimicrobial system is called the LPO system. Glucose oxidase (GO), which is used in the food industry, has been employed as a source of H_2_O_2_ [18].

$$ \mathrm{Glucose} + {\mathrm{O}}_2\overset{\mathrm{GO}}{\to }{\mathrm{H}}_2{\mathrm{O}}_2 + \mathrm{gluconic}\ \mathrm{acid} $$

$$ {\mathrm{H}}_2{\mathrm{O}}_2 + {\mathrm{SCN}}^{\hbox{--}}\overset{\mathrm{LPO}}{\to }{\mathrm{O}\mathrm{SCN}}^{\hbox{--} } + {\mathrm{H}}_2\mathrm{O} $$

A composition containing LPO, GO, glucose, and citrate buffer salts was previously found to exhibit in vitro bactericidal activity in the presence of saliva or SCN^−^ [19]. A preliminary in vivo study suggested that this composition was effective for reducing oral malodor [19]. In a previous clinical trial that enrolled 15 healthy volunteers aged 26–54 years, sucking a trial tablet containing 100 mg of LF, 1.8 mg of LPO, and 24 mg of Sumizyme PGO which was composed of dextrin and GO, had short-term suppressive effects on the concentrations of VSCs in mouth air [20]. The underlying mechanisms of action reducing VSCs were suggested to involve the antimicrobial effects of LF and the LPO system [20] in addition to the inactivating effects of the LPO system on bacterial lyases involved in the production of VSCs [21]. Based on these findings, a powder composition including 20 mg of LF, 2.6 mg of LPO, and 2.6 mg of GO was developed for functional food products [22]. Several commercial food products including this composition were recently launched in Japan.

The aim of the present study was to assess the effects of the single ingestion of a commercial tablet containing 20 mg of LF, 2.6 mg of LPO, and 2.6 mg of GO on oral malodor. We enrolled 40 healthy adults in this trial, which was conducted on a larger scale than a previous related clinical trial [20]. We also investigated the relationships between the treatment effects of the tablet and periodontal conditions of the subjects.

## Methods

### Study food

A commercial tablet (Morinaga Orabarrier, Morinaga Milk Industry Co., Ltd., Tokyo, Japan) was used as the test tablet. The test tablet contained 20 mg of LF, 2.6 mg (≥ 25 units) of LPO, and 2.6 mg (≥ 25 units) of GO as the active ingredients. The test tablet also contained glucose, citric acid, and sodium citrate, which support the effects of the active ingredients. The other ingredients in the test tablet were reduced palatinose, reduced sugar syrup, sorbitol, cellulose, calcium stearate, silicon dioxide, flavor, and sucralose. A placebo tablet contained cornstarch and coloring materials (gardenia pigment) instead of LF, LPO, GO, glucose, citric acid, and sodium citrate. LF (Morinaga Milk Industry Co., Ltd., Tokyo, Japan) and LPO (Tatua, Morrinsville, New Zealand) were purified from bovine milk. GO (Sumizyme PGO, Shin-Nihon Chemical, Aichi, Japan) was composed of dextrin and GO obtained from *Penicillium chrysogenum*. These test and placebo tablets were circular in shape with a diameter of 15 mm and thickness of 6 mm. The tablets were also identical in weight, texture, and appearance, but had slightly different tastes due to their ingredients.

### Study design and subjects

This was a single center, randomized, double-blind, placebo-controlled, crossover study conducted in Japan.

The study was conducted in accordance with Helsinki Declaration of 1975 and as revised in 2013. The study protocol was reviewed and approved by the Research Ethics Committee of Kenshokai (no. OBHALI01, on August 8, 2014). The study was performed at Shimizu Dental Clinic in Takasaki between October 2014 and May 2015 by following the CONSORT guidelines for clinical trial Additional file 1.

Individuals who had been referred to Shimizu Dental Clinic were recruited, and screened after providing written informed consent. Inclusion criteria were all adults aged 20 to 65 who had VSCs greater than the olfactory threshold (H_2_S > 1.5 ng/10 ml and CH_3_SH > 0.5 ng/10 ml air) in mouth air, which were measured using portable gas chromatography as described below. Exclusion criteria were: 1) Subjects with severe liver, kidney, heart, lung, gastrointestinal, blood, endocrine, and metabolic diseases. 2) Subjects receiving treatments for dental diseases such as caries and periodontal disease. 3) Subjects treated with antibiotics in the past month. 4) Subjects who participated in other clinical studies in the past month. 5) Subjects with a history of allergies to milk and/or dairy products. 6) Subjects who were pregnant or lactating, or those who were expecting to become pregnant during the study. 7) Subjects judged inappropriate for the study by the investigator. Subjects were randomly assigned to one of two groups, initially at a 1:1 ratio. On the first test day, subjects in group A (*n* = 20) ingested the test tablet, while subjects in group B (*n* = 20) ingested the placebo tablet. After a 1–4-week washout, each subject ingested the alternative tablet to that on the first test day. No significant changes were made to the methods after trial commencement.

### Outcome and clinical assessments

Oral malodor measurements were conducted in reference to the protocol described previously [23, 24]. On the day of the assessment, each subject was asked to refrain from oral activities including eating, drinking, smoking, and using oral-hygiene practices from the time they woke up until the end of the assessment. Concentrations of VSCs at the baseline were analyzed with a portable gas chromatograph (OralChroma®, FIS Inc., Japan) [24], which was calibrated before starting the study. According to the manufacturer’s instructions, subjects were instructed to keep a disposable plastic syringe in their mouth for 30 s. A sample of their mouth air was taken, and 0.5 ml of sample air was injected into OralChroma. The concentrations of H_2_S and CH_3_SH were measured in each sample. We confirmed correct peak assignments for VSCs in the chromatograms using relevant software (OralChroma data manager, FIS Inc., Japan) [24]. Two OralChroma readings were taken at each time point and the calculated average was recorded as ng/10 ml. The concentration of total VSCs was obtained as the sum of the H_2_S and CH_3_SH concentrations. After the VSC analysis at baseline, each subject was instructed to suck the test or placebo tablet without chewing or swallowing it. The sucking time was measured by the examiner. The concentrations of VSCs were measured 10 and 30 min after ingestion of the tablet.

On the first test day, all subjects were also clinically evaluated for the following periodontal measurements after a series of oral malodor assessments; number of teeth, probing pocket depth (PPD), and bleeding on probing (BOP) [25]. One well-trained examiner performed these assessments. PPD and BOP were entirely measured in each tooth. PPD measurements were recorded as the deepest pocket depth to the nearest millimeter.

The primary endpoint was the concentration of total VSCs at 10 min. The secondary endpoints were the concentrations of H_2_S and CH_3_SH.

### Randomization and blinding procedure

The independent registration center (Morinaga Milk Industry) registered and randomized the subjects. The registration center generated a random sequence using a computer by the permuted block method (block size of 4). The allocation ratio was 1:1. The allocation sequence was concealed from study subjects, the dentist, co-medicals, data manager, and statistical analyst. According to the allocation sequence, the registration center gave a number to the test foods and chronologically assigned subjects to the test food number. After all data were fixed, the allocation sequence was broken.

### Statistical analysis

The sample size was set to 40, which was the maximum possible entry number during the study period. All analyses were based on the intention-to-treat principle. The concentrations of total VSCs, H_2_S, and CH_3_SH did not show a normal distribution and were, thus, transformed logarithmically to approximate a normal distribution and obtain equal variance. We used linear mixed models to analyze data for total VSCs, H_2_S, and CH_3_SH. The fixed effects were treatment, period, and pretreatment values, while the random effect was subjects. The degree of freedom was adjusted by the Kenward-Roger method. We assessed carry-over effects by adding treatment-by-period interactions to the linear mixed model. In the subgroup analysis, we divided subjects into two groups by the median of continuous background data. We used Pearson’s correlation to assess the relationship between the change in total VSCs from the baseline and dental clinical parameters. SAS 9.4 and JMP 9.0 (SAS Institute Inc., North Carolina, US) was used for all analyses. Two-sided *P* < 0.05 was considered significant for all tests.

## Results

**Fig. 1:**
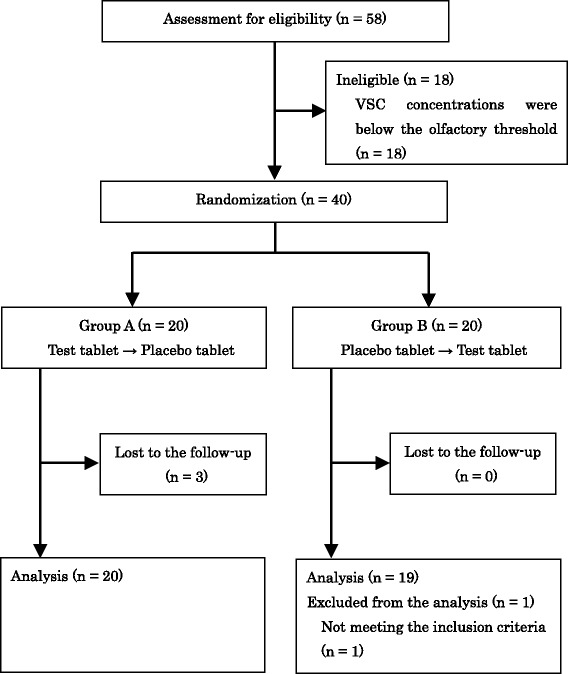
Flow diagram of subjects throughout the study

The flow of our subjects throughout the study is shown in Fig. 1. Fifty-eight males and females were enrolled between October 2014 and May 2015. Forty subjects were randomized to group A (*n* = 20) and group B (*n* =20). After registration of the 40 eligible subjects, enrollment to the study ended in May 2015, and the study was completed in May 2015. Three of the subjects in group A did not complete the study. Two were unable to visit the clinic because of their circumstances. One did not ingest the tablet on the second test day because the investigator judged the concentration of VSCs at the baseline to be insufficient. Therefore, 39 subjects were included in the efficacy analysis based on a full analysis set (FAS). One subject in group B was excluded from the efficacy analysis because the concentration of VSCs at the baseline on the first test day was below the olfactory threshold.

The demographic and baseline characteristics of the full analysis set are listed in Table 1. The mean age was 49.4 years, and 64.1 % were female. The mean number of teeth was 26.9, the average PPD was 4.2 mm, and the average BOP was 15.1 %. The average sucking times in the test and placebo groups were 343.7 ± 118.2 s (179–665 s) and 341.5 ± 130.3 s (190–630 s), respectively. No significant differences were observed in sucking times between the test and placebo tablets.

**Table 1:** Demographic and baseline characteristics of the full analysis set (FAS)

Parameters	Values
Age (mean ± SD)	49.4 ± 15.3
Sex (%)	
Female	64.1 (25/39)
Male	35.9 (14/39)
Clinical parameters (mean ± SD)	
Number of teeth	26.9 ± 2.8
PPD^1^	4.2 ± 0.5
BOP^2^	15.1 ± 20.0

^1^Probing pocket depth (mm)

^2^Bleeding on probing (% of teeth)

### Efficacy

The effects of the test and placebo tablets on the concentrations of total VSCs, H_2_S, and CH_3_SH 10 and 30 min after their ingestion are summarized in Table 2. The concentration of total VSCs at 10 min was significantly lower in the test group than in the placebo group (adjusted difference −0.246 log ng/10 ml; 95 % CI −0.395 to −0.098; *P* = 0.002). No significant differences were observed in the concentrations of total VSCs at 30 min between the test and placebo groups (*P* = 0.199). The concentrations of H_2_S at 10 min were significantly lower in the test group than in the placebo group (adjusted difference −0.349 log ng/10 ml; 95 % CI −0.506 to −0.192; *P* < 0.001). No significant differences were noted in the concentrations of H_2_S at 30 min between the groups (*P* = 0.066). Significant differences were not found in the concentrations of CH_3_SH at 10 and 30 min between the two groups. The differences observed in the concentrations of CH_3_SH between the groups appeared to be slightly higher at 30 min than at 10 min. The concentrations of total VSCs, H_2_S, and CH_3_SH in both groups were significantly lower at 10 and 30 min than at the baseline. The average values (not estimated) of total VSCs, H_2_S, and CH_3_SH are shown in Additional file 2: Figure S1.

**Table 2:** Effects of test and placebo tablets on total VSCs, H_2_S, and CH_3_SH 10 and 30 min after their ingestion

Outcomes	Group	LS mean ± SE^a^	Difference^b^ (95 % CI)	*P*-value
Primary outcome				
Total VSCs				
10 min	Test	0.115 ± 0.078	−0.246	0.002
	Placebo	0.362 ± 0.081	(−0.395 to 0.098)	
30 min	Test	0.173 ± 0.086	−0.136	0.199
	Placebo	0.309 ± 0.090	(−0.348 to 0.075)	
Secondary outcomes				
H_2_S				
10 min	Test	−0.085 ± 0.083	−0.349	< 0.001
	Placebo	0.263 ± 0.086	(−0.506 to 0.192)	
30 min	Test	0.056 ± 0.080	−0.171	0.066
	Placebo	0.226 ± 0.084	(−0.353 to 0.012)	
CH_3_SH				
10 min	Test	−0.635 ± 0.129	−0.044	0.747
	Placebo	−0.591 ± 0.134	(−0.321 to 0.233)	
30 min	Test	−1.070 ± 0.141	−0.265	0.163
	Placebo	−0.806 ± 0.149	(−0.641 to 0.112)	

^a^The concentration (log ng/10 ml) of VSCs estimated using a linear mixed model

^b^Difference (log ng/10 ml) in the LS mean

*LS* least square

No significant interaction was observed between treatment and period (data not shown). This result suggested that carryover effects were not present. The concentrations of VSCs in groups A and B were inversely related between days 1 and 2, showing almost similar changes (Additional file 3: Figure S2).

**Fig. 2:**
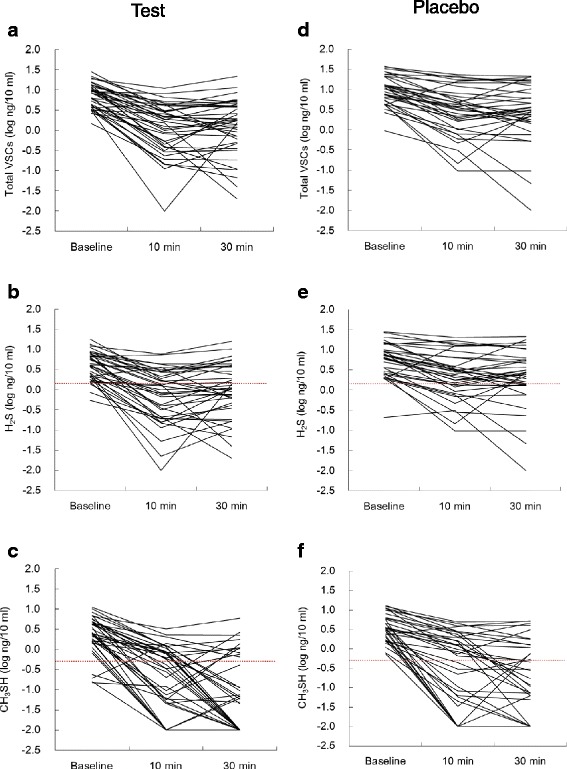
Changes in VSC concentrations in each of the 39 subjects. (**a**, **b**) Total VSCs, (**c**, **d**) H_2_S, and (**e**, **f**) CH_3_SH were measured at the baseline and 10 and 30 min after the ingestion of the test tablet (**a**, **c**, **e**) and placebo tablet (**b**, **d**, **f**). The olfactory thresholds of H_2_S (1.5 ng/10 ml ≈ 0.18 log ng/10 ml) and CH_3_SH (0.5 ng/10 ml ≈ −0.30 log ng/10 ml) are shown in broken lines

Changes in the concentrations of VSCs in each of the 39 subjects examined are shown in detail in Fig. 2. The olfactory thresholds of H_2_S and CH_3_SH were previously reported to be 1.5 and 0.5 ng/10 ml (0.18 and −0.30 log ng/10 ml) mouth air, respectively [26]. The number of subjects with concentrations of H_2_S lower than the olfactory threshold was significantly higher in the test group (61.5 % or 24/39 at 10 min, 59.0 % or 23/39 at 30 min) than in the placebo group (31.4 % or 11/35 at 10 min, 28.6 % or 10/35 at 30 min), according to the chi-squared test (*P* = 0.010 at 10 min, *P* = 0.009 at 30 min). The number of subjects with concentrations of CH_3_SH lower than the olfactory threshold was also higher in the test group (51.3 % or 20/39 at 10 min, 66.7 % or 26/39 at 30 min) than in the placebo group (42.9 % or 15/35 at 10 min, 51.4 % or 18/35 at 30 min), and no significant differences were observed between the groups at 10 min (*P* = 0.468) or 30 min (*P* = 0.183). The percentages of subjects with concentrations of CH_3_SH lower than the detection limit (=0 ng/10 ml) were 17.9 % (7/39) at 10 min and 43.6 % (17/39) at 30 min in the test group, and 20.0 % (7/35) at 10 min and 25.7 % (9/35) at 30 min in the placebo group, respectively.

**Fig. 3:**
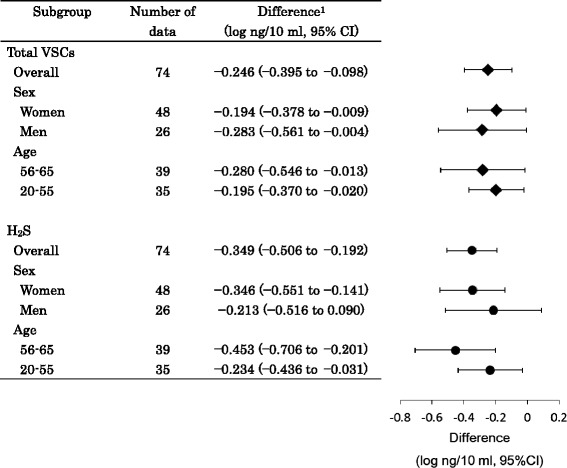
Subgroup analyses for primary and secondary outcomes. ^1^Difference between the test and placebo groups in the extent of reductions in the concentration of total VSCs from the baseline to 10 min after the ingestion of tablets

The results of the subgroup analysis for outcomes are shown in Fig. 3. The difference in the reduction of VSCs from the baseline to 10 min between the test and placebo groups was adopted as the treatment effect. The treatment effect on total VSCs and H_2_S did not significantly differ when subjects were fractionated by sex and age.

The results of a correlation analysis among the effects on total VSCs, PPD, BOP, and age of subjects are shown in Table 3. The reducing effect on total VSCs positively correlated with PPD (*r* = 0.358, *P* = 0.035). A correlation was not detected between the treatment effect on total VSCs and BOP. Age positively correlated with PPD (*r* = 0.603, *P* < 0.001), whereas no correlation was observed for age and the treatment effect on total VSCs.

**Table 3:** Pearson correlations between effects on total VSCs and parameters for the clinical assessment

Parameter	vs. Parameter	*r*	*P*-value
PPD^a^	ΔΔTotal VSCs^b^	0.358	0.035
BOP^c^	ΔΔTotal VSCs	0.273	0.113
Age	ΔΔTotal VSCs	0.200	0.249
Age	PPD	0.603	< 0.001

^a^The mean value of the probing pocket depth (mm) of each tooth

^b^Reducing effect on total VSCs; Differences between the test and placebo groups in the extent of reductions in the concentration of total VSCs from the baseline to 10 min after ingestion of the tablets

^c^Bleeding on probing (% of teeth)

We investigated the influence of the sucking time of the test tablet on suppressing the concentration of VSCs by adding a treatment-by-sucking time interaction to the linear mixed model. Regarding the concentration of total VSCs at 10 min, the interaction was not significant (*P* = 0.196).

### Safety

General conditions were obtained by interviewing subjects on each day of the assessment. One subject in the placebo group had cold-like symptoms during one of the washout periods, which did not appear to be related to any treatment administered in the present study. Thus, no adverse events related to the treatment were observed in any of the 40 subjects during the study.

## Discussion

This randomized clinical trial was designed to evaluate the efficacy of the single ingestion of a commercial tablet containing 20 mg of LF, 2.6 mg of LPO, and 2.6 mg of GO on oral malodor. Our results demonstrated that oral malodor in the general population was lower following the single ingestion of a tablet containing LF, LPO, and GO than a placebo tablet. As for the primary outcome, the concentration of total VSCs at 10 min was significantly lower in the test group than in the placebo group. Furthermore, similar results were obtained for the concentrations of H_2_S and total VSCs. It was estimated that the test tablet reduced the concentrations of total VSCs and H_2_S in the mouth by approximately 57 % (≈10^–0.246^) and 45 % (≈10^–0.349^) that of the placebo tablet, respectively. No significant differences were observed in the concentrations of CH_3_SH at 10 and 30 min between the groups; however, the difference noted in the concentration of CH_3_SH at 30 min appeared to be slightly greater than that at 10 min. The proportion of CH_3_SH to total VSCs was smaller than that to H_2_S, and may be one of the reasons why a significant difference was not noted in CH_3_SH concentrations. The test and placebo tablets both significantly reduced the concentration of VSCs at 10 and 30 min to lower than that at the baseline. The suppressive effects of the placebo tablet may also have been one of the reasons why significant differences were not observed between the two groups at 30 min.

VSCs are produced from sulfur-containing amino acids by oral anaerobes. The effects of many antimicrobial agents on oral malodor have been reported previously [5]. LF and the LPO system, consisting of LPO, GO, glucose, and SCN^−^, exert in vitro antibacterial effects against oral pathogens [12, 19]. We consider these antibacterial effects to be weaker than those of antibiotics which might affects indigenous bacteria and cause superinfections. In a previous clinical trial, terminal restriction fragment length polymorphism findings suggested that a tablet containing LF, LPO, and GO reduced one fragment assigned to bacterial species including VSC-producing bacteria [20]. In spite of this moderate antibacterial effect, the suppressive effects of the active ingredients on VSCs were demonstrated in the present and previous studies. A recent in vitro study reported that the LPO system exhibited inactivation activity against the bacterial lyases related to the production of VSCs [21]. Inactivation activity may be the main contributor to suppressive effects on VSCs. Thus, the test tablet immediately exerted suppressive effects on oral malodor. The reproduction of lyases by viable bacterial cells may gradually weaken suppressive effects.

Previous studies reported that a tablet containing LF, LPO, and GO significantly suppressed the concentration of VSCs 2 h after its ingestion to less than that at the baseline [20, 22]. In the present study, the concentration of total VSCs in the test group was constantly low from 10 min to 30 min. The concentrations of H_2_S and CH_3_SH were lower than the olfactory threshold at 10 min in more than half of the subjects in the test group. The percentage of subjects in the test group with concentrations lower than the olfactory threshold at 30 min was nearly the same as that at 10 min. These results suggest that the suppressive effects of the tablet containing LF, LPO, and GO on oral malodor persisted for some time.

The test tablet contained glucose, citric acid, and sodium citrate, which support the effects of the active ingredients. Glucose, citric acid, and sodium citrate were unlikely to have exerted suppressive effects on VSCs because they are known not to exhibit antibacterial activity against periodontal bacteria [19] or inactivation effects on bacterial lyases involved in the production of VSCs [21]. The placebo tablet contained cornstarch and coloring materials (gardenia pigment), which were added as replacements. Although digested starch may be a source of a cariogenic biofilm, previous in vitro oral biofilm and in vivo studies reported that starch was markedly less cariogenic than sucrose [27, 28]. Furthermore, we considered the influence of cariogenic biofilm formation on VSC production to have been negligible in the short term in the present study because biofilm formation requires a long period of time and most cariogenic species do not play a role in the production of VSCs [2]. The placebo tablet exerted some suppressive effects on oral malodor. To the best of our knowledge, the suppressive effects of the ingredients of the placebo tablet including cornstarch and coloring materials on oral malodor have not been reported previously. On the other hand, many studies demonstrated that tongue cleaning reduced the amount of tongue coating and number of oral bacteria, thereby effectively improving oral malodor [2, 5, 29]. Another previous clinical trial suggested that a placebo tablet without active ingredients reduced VSCs in the short term through its mechanical cleaning effect [30]. Therefore, the suppressive effects of the placebo tablet on oral malodor in the present study may have been due to its mechanical cleaning effects. We assumed that the test tablet suppressed VSCs not only through the antimicrobial activities of LF and the LPO system against VSC-producing bacteria, but also by mechanical cleaning during sucking.

The concentration of total VSCs was significantly lower 10 min after ingestion of the previous trial tablet, which contained 100 mg of LF, 1.8 mg of LPO, and 24 mg of Sumizyme PGO, than the placebo tablet [20]. In the present study, the concentration of VSCs was significantly lower 10 min after ingesting the commercial tablet containing 20 mg of LF, 2.6 mg of LPO, and 2.6 mg of GO than the placebo tablet. These results suggested that the efficacy of this commercial tablet, which had less LF, in suppressing oral malodor was similar to that of the previous trial tablet in the short term.

A limitation of our study is the slight difference in taste between the tablets, which was due to their ingredients. Although our subjects may have been able to distinguish between these tablets, they were unable to control the concentrations of VSCs measured by OralChroma. Therefore, we considered the robustness against a measurement bias to have been sustained.

The treatment effects of the test tablet on total VSCs and H_2_S did not differ when subjects were fractionated by sex and age. Furthermore, a correlation was not observed between age and the treatment effect on VSCs. A previous survey reported no significant differences in VSCs between males and females or among ages [31]. It appears reasonable to extrapolate the results obtained in the present study to general populations.

The reducing effect on total VSCs positively correlated with PPD. These results suggested that treatment effects were greater in subjects with deep periodontal pockets. Tanaka et al. found positive coefficients between the percentages of probing pocket depth ≥ 4 mm and the population of periodontal pathogens including *P. gingivalis* on the tongue dorsum, and the population of these pathogens positively correlated with the concentration of total VSCs [32]. In the present study, subjects with deep pocket depths may have had a larger population of VSC-producing bacteria, which are considered the target of LF and the LPO system.

In order to allow active ingredients to function effectively in the mouth, subjects were asked to suck the tablets without biting or swallowing them. The time for tablets to dissolve completely was approximately 3 to 11 min. We investigated the influence of the sucking time of the test tablet on suppressing the concentration of VSCs. The sucking time did not interact with the treatment effects. These results suggested that the suppressing effects on oral malodor occurred when the subject sucked the test tablet for at least 3 min. In a previous in vitro study, a composition containing LPO, GO, glucose, and SCN^−^ reduced the number of *A. actinomycetemcomitans* by more than 1 log unit after 3.75 min [19], and was found to inactivate the bacterial lyase related to VSC production after 10 min [21]. These in vitro bactericidal and inactivating effects in a short time period may contribute to the immediate effects of the test tablet on oral malodor.

No adverse events related to the treatment were observed in any of the 40 subjects during the study. All ingredients in the current test tablet including LF, LPO, and GO have been permitted as a food or food additive in Japan. Furthermore, the long-term ingestion of previous trial tablets containing LF (300 mg/day), LPO (5.4 mg/day) and Sumizyme PGO (72 mg/day) for 12 weeks had no adverse events in general or on the oral condition [33]. Therefore, the test tablet in the present study may be continuously and safely taken on a daily basis.

Severe or long-standing oral malodor has a negative impact on self-confidence and social interactions [1]. Therefore, a daily treatment for oral malodor is considered important. A previous study demonstrated that VSC concentrations decreased after a meal and then gradually increased between meals [23]. Since the test tablet has excellent portability and immediately suppresses oral malodor, it has potential as a daily treatment, particularly for suppressing VSCs between meals. Furthermore, VSCs have been shown to affect the progression of periodontal disease [5–7]. The long-term administration of this tablet may potentially contribute to maintaining the oral hygiene status.

## Conclusions

The results of the present clinical trial suggested that the single ingestion of a tablet containing 20 mg of LF, 2.6 mg of LPO, and 2.6 mg of GO exhibited suppressive effects on VSCs in mouth air in the general population.

## Additional files

Additional file 1: CONSORT checklist items. (DOC 218 kb) Additional file 2: Figure S1. Effects of test tablets on the concentration of VSCs in mouth air. (A) Total VSCs, (B) H_2_S, and (C) CH_3_SH were measured at the baseline and 10 and 30 min after the ingestion of tablets. Data represent the mean ± SD. The olfactory thresholds of H_2_S (1.5 ng/10 ml ≈ 0.18 log ng/10 ml) and CH_3_SH (0.5 ng/10 ml ≈ −0.30 log ng/10 ml) are shown in broken lines. **: Significant differences between the groups (*P* < 0.01). (DOCX 83 kb) Additional file 3: Figure S2. Effects of test tablets on the concentration of VSCs categorized by the allocation group. Subjects in group A (closed circle) ingested the test tablet on the first test day and the placebo tablet on the second test day, while subjects in group B (open triangle) ingested the placebo tablet on the first test day and the test tablet on the second test day. (DOCX 97 kb)
