# Supplementary figures and images for: A randomized, double-blind, crossover, placebo-controlled clinical trial to assess effects of the single ingestion of a tablet containing lactoferrin, lactoperoxidase, and glucose oxidase on oral malodor
Source: BMC Oral Health. 2016 Mar 22;16:37. doi: 10.1186/s12903-016-0199-7 (PMC4802841; doi:10.1186/s12903-016-0199-7)

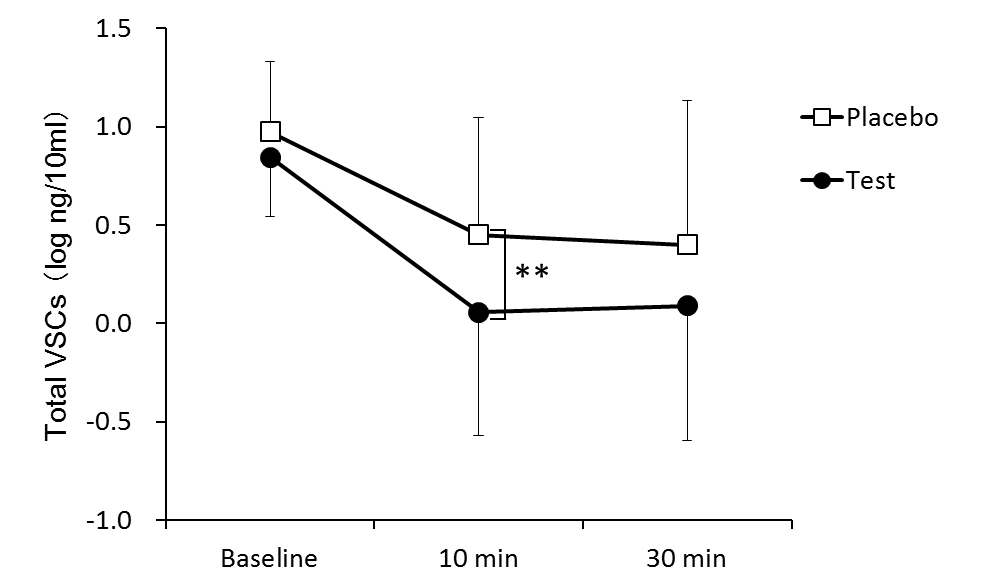

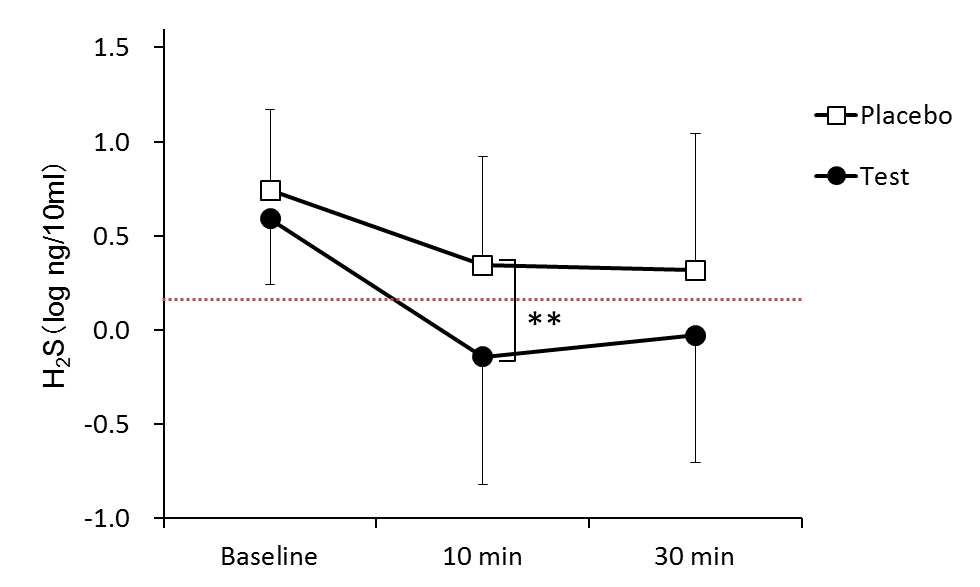

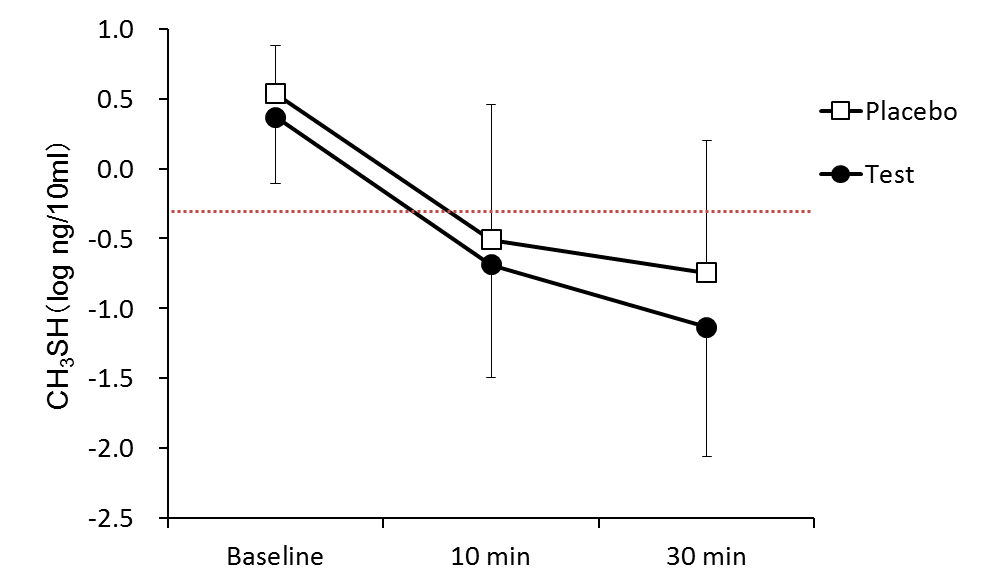


C

B

A

Supplemental Figure 1

Supplement: Additional file 2: Figure S1. — Effects of test tablets on the concentration of VSCs in mouth air. (A) Total VSCs, (B) H2S, and (C) CH3SH were measured at the baseline and 10 and 30 min after the ingestion of tablets. Data represent the mean ± SD. The olfactory thresholds of H2S (1.5 ng/10 ml ≈ 0.18 log ng/10 ml) and CH3SH (0.5 ng/10 ml ≈ −0.30 log ng/10 ml) are shown in broken lines. **: Significant differences between the groups (P < 0.01). (DOCX 83 kb) [file 12903_2016_199_MOESM2_ESM.docx]

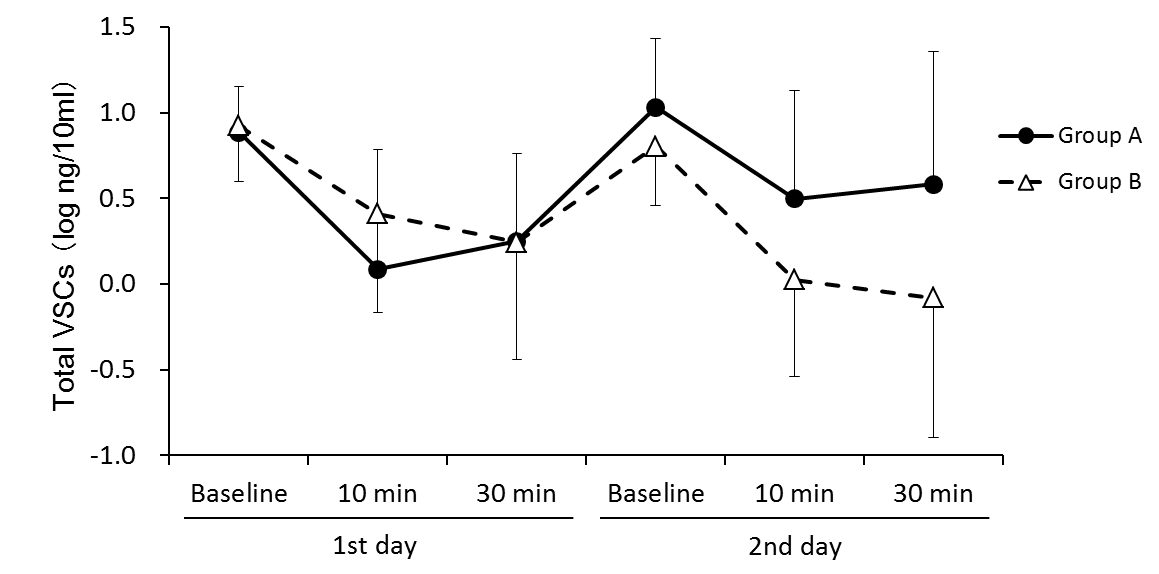


A


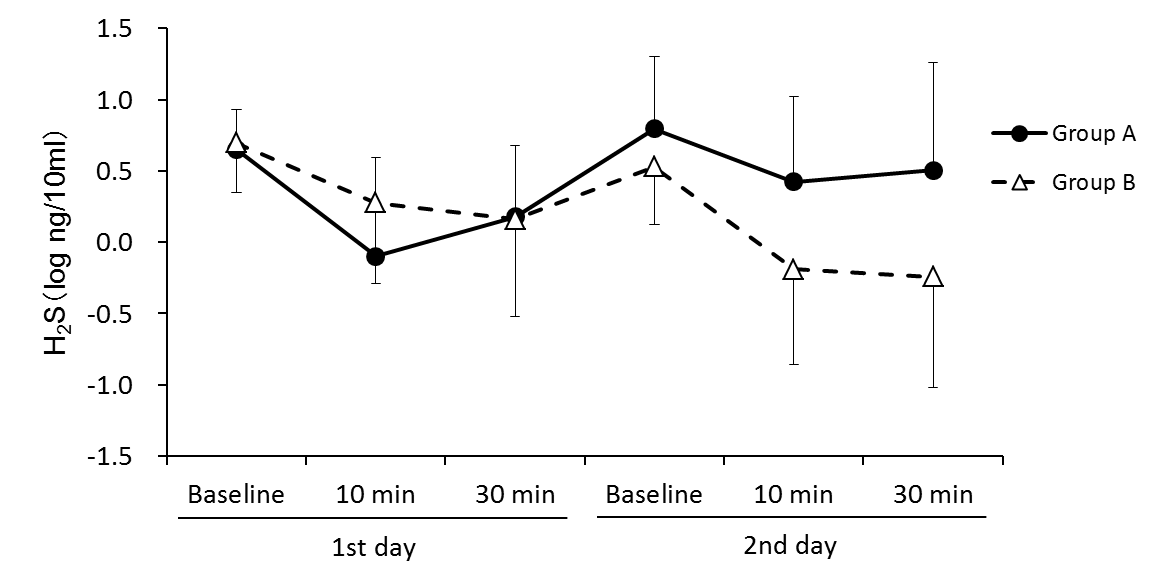


B


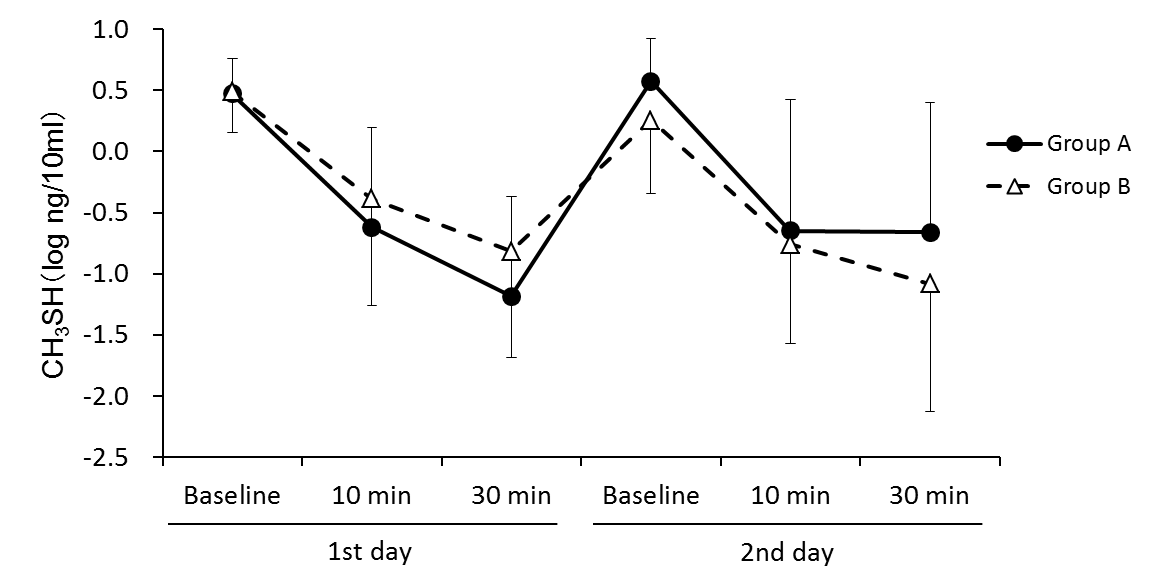


C

Supplemental Figure 2

Supplement: Additional file 3: Figure S2. — Effects of test tablets on the concentration of VSCs categorized by the allocation group. Subjects in group A (closed circle) ingested the test tablet on the first test day and the placebo tablet on the second test day, while subjects in group B (open triangle) ingested the placebo tablet on the first test day and the test tablet on the second test day. (DOCX 97 kb) [file 12903_2016_199_MOESM3_ESM.docx]
